# Impact of UV‑C Light Treatment on the Vitamin D Content and Quality of Bread

**DOI:** 10.1021/acsomega.5c03225

**Published:** 2025-07-30

**Authors:** Edanur Kömürlü, Gurbuz Gunes, Aylin Met Özyurt

**Affiliations:** † Beko, Research & Development Center, Istanbul 34950, Turkey; ‡ Department of Food Engineering, Faculty of Chemical and Metallurgical Engineering, 52971 Istanbul Technical University, Maslak, Istanbul 34467, Turkey

## Abstract

Vitamin D deficiency is a global health concern linked to various chronic diseases. Although food fortification is a promising solution, its implementation remains limited by processing losses, matrix inconsistency, and clean-label trends. Certain foods, including mushrooms and yeast-containing bakery products, naturally contain provitamin D_2_ (ergosterol), which can be photoconverted into vitamin D_2_ by ultraviolet (UV) light. While UV-based vitamin D_2_ enrichment has been extensively studied in mushrooms, its application in bread remains largely unexplored. This study investigated UV-C irradiation as a biofortification strategy for white and whole wheat bread, evaluating the effects of UV-C dose (0.50 and 2.00 kJ/m^2^) and dose rate (0.03 and 0.13 kJ/m^2^·min) on vitamin D_2_ content and bread quality attributes. Optimized UV-C parameters enabled a novel strategy to increase vitamin D_2_ content in bread without compromising the quality. UV-C treatments resulted in varying vitamin D_2_ levels in bread, depending on the dose and dose rate, whereas control bread did not contain any detectable vitamin D_2_. The highest vitamin D_2_ content was obtained when the 2.00 kJ/m^2^ dose was applied at 0.03 kJ/m^2^·min dose rate, although this treatment altered odor and taste. However, other treatments increased vitamin D_2_ without compromising the quality. The optimal treatment of 2.00 kJ/m^2^ dose at 0.13 kJ/m^2^·min dose rate achieved the best balance between vitamin D_2_ enrichment and sensory acceptability. Under the optimal conditions, a 14 g serving of UV-C treated white bread and a 15.5 g serving of whole wheat bread provided 27 and 37% of the recommended dietary allowance (RDA) for vitamin D, respectively. Thus, UV-treated bread represents a valuable and practical source of plant-based vitamin D for consumers, particularly for vegetarians or vegans.

## Graphical abstract

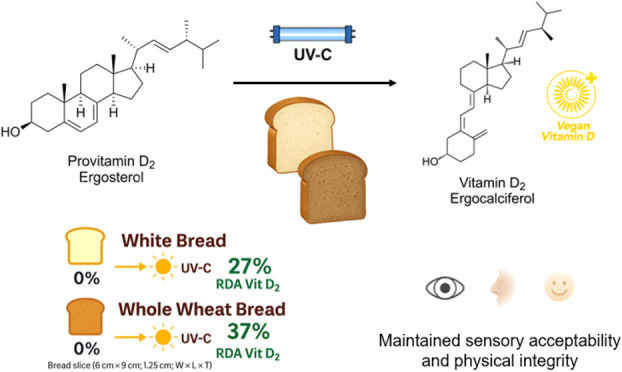

## 1 Introduction

Vitamin D is an essential nutrient that plays a critical role in maintaining bone health by regulating calcium–phosphorus metabolism, supporting neuromuscular and immune function, and preventing osteoporosis and osteomalacia.^1^ Additionally, it has been associated with the prevention of several chronic diseases, including cardiovascular disease, multiple sclerosis, diabetes, and certain cancers.^2,3^ Adequate vitamin D levels may also support mental health by alleviating symptoms of depression and contribute to improved metabolic outcomes by reducing the risk of obesity.^4,5^ Furthermore, emerging evidence suggests a potential protective role of vitamin D in infectious diseases, particularly coronavirus disease (COVID-19), with studies indicating that individuals with deficient or insufficient vitamin D status are more likely to experience severe symptoms.^6,7^ Despite its broad physiological benefits, vitamin D deficiency is a global and growing issue in human nutrition and health. This is largely attributed to the limited availability of natural dietary sources and the decreased ability of the skin to produce vitamin D due to insufficient sun exposure, which is influenced by geographic location, seasonal variation, lifestyle, skin pigmentation, and age.^8^ Individuals following strict vegan or vegetarian diets are particularly at risk of vitamin D deficiency since natural dietary sources of vitamin D in plant-based foods are scarce. The primary dietary sources of vitamin D include animal-based foods such as fatty fish (e.g., salmon, herring, and sardines), cod liver oil, and egg yolks. Certain mushrooms represent one of the few plant-based sources of vitamin D, but they provide it in only small quantities.^9^ Due to the few natural sources, it is difficult for the human diet to supply a sufficient amount of vitamin D.

Food-based strategies have become essential to improve vitamin D levels across populations due to certain limitations. One effective approach has been fortification, which involves adding vitamin D to widely consumed foods, including milk, yogurt, fruit juices, bread, and breakfast cereals. This method has been successful in increasing serum 25-hydroxyvitamin D [25­(OH)­D] concentrations.^10^ However, this strategy is often constrained by several challenges, such as reduced stability during thermal processing and storage, uneven distribution within the food matrix, and concerns about maintaining a clean-label product.^11^ While supplementation is a common alternative, its effectiveness may be limited by inconsistent intake, and the use of animal-derived vitamin D_3_ makes it unsuitable for vegan or plant-based diets.^12^

As an alternative to conventional fortification, biofortification through ultraviolet (UV) light exposure has emerged as a natural and potentially sustainable approach. This method increases vitamin D levels intrinsically within the food matrix by converting precursor molecules into active forms of vitamin D through photoconversion. Ergosterol, a provitamin D_2_ naturally found in fungi, has been extensively studied in mushrooms, where UV exposure promotes its conversion to vitamin D_2_.^13^ Expanding this approach to yeast-containing food products represents a promising strategy for enhancing natural vitamin D_2_ levels. *Saccharomyces cerevisiae*, commonly known as baker’s yeast, contains considerable amounts of ergosterol within its cell membrane.^14^ Therefore, yeast-leavened bread products can be considered suitable candidates for UV-based biofortification. Although limited studies have evaluated bread and baker’s yeast as sources of vitamin D_2_, the European Food Safety Authority (EFSA) has confirmed that both UV-treated bread and yeast enriched with vitamin D_2_ are safe for consumption.^15,16^

Despite these advancements, the application of a UV-induced biofortification strategy in bread products is still largely unexplored, particularly concerning treatment variables such as dose and dose rate. The objective of this study was to investigate the impacts of UV-C treatments on the vitamin D content and key quality attributes of both white and whole wheat bread crumbs. The findings from this study could contribute to public health by introducing a practical, plant-food-based strategy that enhances dietary vitamin D intake while maintaining a clean label.

## 2 Materials and Methods

### 2.1 Bread Samples and Reagents

Two types of bread were purchased from the Istanbul Halk Ekmek (IHE) store, including sliced white bread and sliced whole wheat bread. The ingredients of white bread as stated in its label were: Wheat flour, water, gluten, bread yeast, sunflower oil, whey powder, salt, vegetable emulsifier (sodium stearoyl-2-lactylate), food enzymes (α-amylase, hemicellulase), antioxidant (ascorbic acid), anticaking agent (calcium carbonate), and preservative (calcium propionate). The ingredients of whole wheat bread were: 100% whole wheat flour, water, bread yeast, gluten, salt, rye sourdough powder, whey powder, grape vinegar, food enzymes (hemicellulase, lipase, α-amylase, glucose oxidase), and preservative (calcium propionate).

Methanol (CH_4_O, ≥99.8%), *n*-hexane (C_6_H_14_, ≥98%), potassium hydroxide (KOH, ≥85.0%), l­(+)-Ascorbic Acid (C_6_H_8_O_6_, ≥99%), tetrahydrofuran (C_4_H_8_O, ≥99%), ethanol (C_2_H_6_O, ≥99.9%), and HPLC-grade purified water were purchased from Merck (Darmstadt, Germany). The Ergocalciferol (C_28_H_44_O) analytical standard was purchased from Sigma-Aldrich (St. Louis, MO).

### 2.2 Treatment of Breads and Exposure Design

To ensure uniformity, the crust section of the bread slices was removed and only the crumb was used for treatments and analysis. Bread crumb slices were cut into dimensions of 6 × 9 cm^2^ (width × length). The thickness of the sliced samples was 1.25 cm. This part was preferred due to its homogeneous porous structure, which allows consistent UV-C penetration and minimizes structural variability across samples.

The UV-C irradiation of the samples was performed in a custom-made chamber using a monochromatic UV lamp emitting UV-C light at 254 nm, with a lamp power range of 0.5–1.2 W and a lamp voltage range of 150–200 V. Samples were placed on a tray positioned under the lamp at a fixed distance of 10 cm. The chamber temperature was maintained at 0.9 ± 0.3 °C. The irradiation zone was defined as a 6 cm × 9 cm area, corresponding to the dimensions of the bread crumb slices used in the experiment. To characterize the irradiance distribution, the exposure zone was subdivided into a grid of 3 cm × 3 cm squares. A digital radiometer (HD2102.1, Delta Ohm, Italy) was used to measure irradiance (μW/cm^2^) at eight points within each of two target regions - six peripheral and two central. Based on the average irradiance values, two regions with distinct dose rates were identified: a low-dose-rate region (0.03 kJ/m^2^·min) and a high-dose-rate region (0.13 kJ/m^2^·min). The samples were placed in these positions for irradiation at two different dose rates, as specified in Table 1. The control samples were also kept in the treatment chamber for the longest exposure time while the UV-C lamp was off. After exposures, all bread samples were analyzed directly without post-treatment storage.

**Table 1:** Exposure Time to Achieve the Target Dose Levels

	dose (kJ/m^2^)	
UV-C dose rate	0.50	2.00
low (0.03 kJ/m^2^·min)	15 min	64 min 21 s
high (0.13 kJ/m^2^·min)	3 min 30 s	15 min

The UV-C treatments were divided into three experiment groups based on the type of analysis to be performed: chemical (vitamin D_2_ and moisture), physical (color and texture), and sensory evaluation (Figure 1). Samples in each group were irradiated under consistent conditions, and the samples were then analyzed separately.

**Figure 1:**
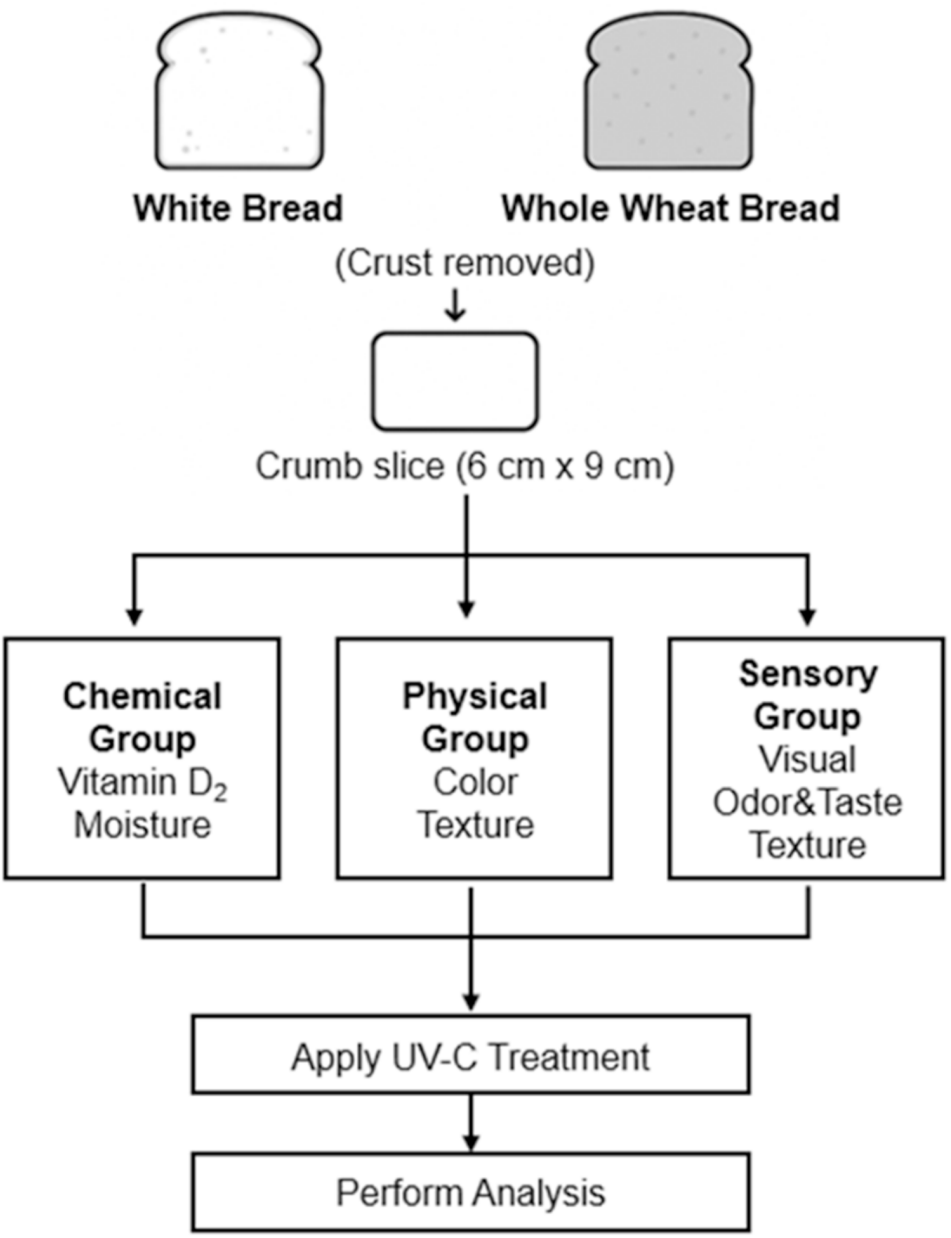
Flowchart of the experimental procedure.

### 2.3 Analysis of Vitamin D_2_ Content

The vitamin D_2_ contents of the samples were measured according to the method of Pedrali et al.^17^ with minor modifications. Homogenized bread samples (10 g) were weighed into a 250 mL flask and mixed with 1 g of l-ascorbic acid, 50 mL of ethanol, and 25 mL of 50% potassium hydroxide. The mixture was heated at 85 °C for 30 min and then cooled to an ambient temperature before being extracted twice with 10 mL of deionized water and 30 mL of *n*-hexane in a separating funnel. The pooled organic phase was washed three times with deionized water. Then, the organic phase was poured into a flask and evaporated to dryness with a 180 mbar vacuum at 40 °C in a rotary evaporator (Buchi, R300). The dried extract was recovered by washing the flask with a 1 mL tetrahydrofuran:methanol:water (67:27:6, v/v/v) mixture twice and filtered using a 0.45-μm PTFE membrane prior to HPLC analysis.

The filtered sample was analyzed using an HPLC-DAD instrument (Shimadzu, Prominence-i LC-2030C 3D Plus, America) at a wavelength of 264 ± 4 nm. The analysis was performed using a reverse-phase C18 column (InertSustain, 4.6 mm × 250 mm × 5 μm) at 25 °C with a mobile phase of tetrahydrofuran:methanol:water (67:27:6, v/v/v) at an injection volume of 20 μL and a flow rate of 0.5 mL/min. The vitamin D_2_ content was quantified based on the calibration curve prepared by the vitamin D_2_ standard (Sigma–Aldrich, E0900000). Vitamin D_2_ analysis was conducted on three independently replicated samples.

### 2.4 Determination of Moisture Content

The moisture contents of bread slices were determined by the air-oven method according to Sehn and Steel.^18^ Approximately 3 g of each sample was weighed and dried in an oven at 105 °C until a constant weight was reached. The moisture content was calculated based on the difference between the initial and final weights. Moisture analysis was conducted on three independently treated samples.

### 2.5 Texture Analysis

Crumb pieces of 2.5 cm × 2.5 cm × 1.25 cm (length × width × height) were prepared and used for texture analysis (TA.XTplus100C, Stable Micro System, U.K.), as described by Tauferová et al.^19^ Two pieces of the samples stacked on top of each other were compressed to 40% of the initial height with a 36 mm diameter cylindrical probe (P/36R). The test speed was 1.7 mm/s, and the trigger force was 0.05 N. The maximum force measured was designated as the crumb firmness. Texture analysis was conducted on five independently treated samples.

### 2.6 Color Analysis

Bread crumb color was described using the total color change (Δ*E*), chroma (C*), and hue angle (h*). The color values (L*, a*, b*, C*, h*) of the bread crumbs were measured using a colorimeter (Konica Minolta CM-5, Japan) with a 30 mm target mask.^20^ The color difference values were calculated by the equations below:

$$\Delta E=\sqrt{\left[{{(L}^{*}-L_{0}^{*})}^{2}+{{(a}^{*}-a_{0}^{*})}^{2}+{{(b}^{*}-b_{0}^{*})}^{2}\right]}$$

where Δ*E* was the total color change; was calculated in comparison to the control bread samples based on the changes in L*, a*, and b* values (ΔL*, Δa*, and Δb*). The L*, a*, and b* values of the UV-treated bread samples were measured after UV-C treatment, while those of the control samples were measured after the longest duration in the chamber (*L*_0_*, *a*_0_*, and *b*_0_*). Color analysis was conducted on five independently treated samples.

### 2.7 Sensory Evaluation

Sensory attributes of bread samples, including visual appearance, odor and taste, and texture profile were evaluated using a 0–5 scale.^21^ Each sample was labeled with a random three-digit code. During visual evaluation, the color and homogeneity of the crumbs were assessed. The evaluation of odor and taste included the identification of any foreign odors, aroma, and foreign tastes; the texture evaluation focused on the softness and chewiness of the bread crumb. Following these assessments, the overall acceptance of the bread samples was determined. At each session, panelists were provided with untreated reference bread to consider before evaluating the samples. The sensory evaluation was conducted by a panel of 10 trained panelists, who assessed treated samples in triplicate across three separate sessions. This panel size falls within the generally accepted range of 8–12 panelists for descriptive sensory analysis, ensuring the reliability of the results.^22^

### 2.8 Statistical Analysis

All results are reported as means ± standard deviations (SD). The data were subjected to analysis of variance (ANOVA) and Tukey’s tests with a 95% confidence level using statistics software (Minitab 18, Minitab Inc., Pennsylvania).

## 3 Results and Discussion

### 3.1 Vitamin D_2_ Content

Effects of UV-C treatment on the vitamin D_2_ content of the bread slices, reported on a dry basis, are given in Table 2. Before UV-C radiation, the control bread samples contained less than the limit of quantification (LOQ: 0.11 μg/g) for vitamin D_2_. However, the UV-C treatments resulted in a significant increase in the vitamin D_2_ content in these samples. While control bread samples without UV-C treatment had no vitamin D, different levels of vitamin D_2_ were obtained in the UV-C-treated bread samples depending on the UV-C dose and dose rate (*p* < 0.05). The vitamin D_2_ levels showed a positive correlation with the UV-C dose and a negative correlation with the UV-C dose rate.

**Table 2:** Effect of UV-C Treatment Conditions on the Vitamin D_2_ Content (μg/g Based on Dry Weight) of the Bread Samples^a^

dose (kJ/m^2^)	dose rate (kJ/m^2^·min)	white bread	whole wheat bread
0 (control)	0.00	not detected^e^	not detected^e^
0.50	0.03	0.41 ± 0.03^c^	0.51 ± 0.03^c^
	0.13	0.25 ± 0.03^d^	0.31 ± 0.05^d^
2.00	0.03	0.57 ± 0.03^a^	0.82 ± 0.06^a^
	0.13	0.50 ± 0.03^b^	0.64 ± 0.02^b^

a Values with different lowercase letters in the same column are significantly different (*p* < 0.05). Values are the means ± standard deviations of three observations.

The conversion of ergosterol to vitamin D_2_ is highly dependent on the UV-C energy applied. A higher dose of UV-C (2.00 kJ/m^2^) resulted in an increased vitamin D_2_ content. Similar to our findings, a recent study demonstrated that prolonging UV-B exposures from 0 to 3 h in various mushroom species, by using an 18 W UV-B lamp positioned 15 cm above the samples, significantly increased vitamin D_2_ synthesis, indicating a dose-dependent relationship between UV treatment and vitamin D_2_ formation across different food matrices.^23^ In another study on mushrooms, extending UV-B exposure time from 20 to 60 min at a fixed intensity of 2.00 W/cm^2^ (equivalent to 24,000–72,000 kJ/m^2^) significantly increased vitamin D_2_ content, while further increases in dose resulted in a gradual decline, likely due to photodegradation.^24^ Although our study utilized lower doses, we observed that vitamin D_2_ levels increased with dose, suggesting that UV-C exposure remained within an efficient linear conversion range. Roberts et al.^25^ investigated the effects of both UV-B dose and dose rate on vitamin D_2_ synthesis in mushrooms. They also found that increasing the UV-B dose (0.5, 1.0, and 1.5 J/cm^2^) resulted in increased synthesis of vitamin D_2_. However, they noted no effects of the dose rates (0.46, 0.75, and 1.0 mW/cm^2^) on vitamin D_2_ concentration at 0.5 J/cm^2^ and 1.5 J/cm^2^ doses, while the higher dose rates led to higher concentrations of vitamin D_2_ at 1.0 J/cm^2^ UV-B dose. This is contrary to our observations and may be related to different wavelengths and much lower dose rates applied in our study. We observed that a lower UV-C dose rate of 0.03 kJ/m^2^·min (equivalent to 0.05 mW/cm^2^) resulted in more vitamin D_2_ synthesis in bread compared to the 0.13 kJ/m^2^·min (equivalent to 0.22 mW/cm^2^) dose rate. The lower dose rate in our study may have contributed to the higher vitamin D_2_ content during extended exposure times.

The 2015 EFSA report reviewed data submitted by Viasolde AB, a Swedish company specializing in UV light technology to enhance the vitamin D content in bread. This was part of their application for the approval of UV-treated bread as a novel food.^16^ The submitted data included information about the vitamin D_2_ content in the crusts of both white and rye bread after UV exposure. In samples that were not exposed to UV radiation, no vitamin D_2_ was detected, which aligns with our findings. When a dose of 9 mJ/cm^2^ (equivalent to 0.09 kJ/m^2^) of UV-C (254 nm) was applied to the crust of white bread, the resulting vitamin D_2_ contents were found to be between 2.5 and 2.7 μg/100 g, which is lower than our results. The authors did not specify the dose rate/light intensity or treatment time in the study, but the applied dose was lower than those used in our research. Moreover, UV radiation may have been less effective in vitamin D_2_ formation in the crust compared with treatment applied on the crumb of the bread.

In all UV-C treatments of our study, whole wheat bread exhibited a higher vitamin D_2_ content than white bread (*p* < 0.05). The highest amount of vitamin D_2_ was obtained with a high UV-C dose of 2.00 kJ/m^2^ at a low-dose rate of 0.03 kJ/m^2^·min, yielding 0.57 μg/g in white bread and 0.82 μg/g on a dry weight basis in whole wheat bread. The interaction between dose and dose rate was significant (*p* < 0.05) for the vitamin D_2_ content of white bread, but not significant (*p* > 0.05) for the whole wheat bread. In white bread, at a UV-C dose of 0.50 kJ/m^2^, the effect of the dose rate was more pronounced, with the low-dose rate leading to a higher vitamin D_2_ content compared to the treatment at 2.00 kJ/m^2^.

While the ergosterol content was not quantified in this study, all bread samples were purchased from the same production batches to minimize variability in ergosterol levels due to differences in raw material. The stimulation of vitamin D_2_ formation in bread samples after UV-C treatment is believed to be related to their ergosterol content, primarily derived from bakers’ yeast. Besides, research has shown that the bran contains higher levels of ergosterol compared to flour.^26^ Whole wheat flour includes bran, which is absent in the flour used for white bread. As a result, additional ergosterol from the bran may contribute to the higher vitamin D_2_ levels observed in the UV-C-treated whole wheat bread. Thus, the difference in vitamin D_2_ content between the two bread types could be influenced by the inherent differences in their precursor levels.

Additionally, moisture content has been shown to affect the photoconversion efficiency of ergosterol to vitamin D_2_, with optimal conversion reported within a moisture range of 70–80% as noted in mushrooms.^13^ In our study, the moisture content in the whole wheat bread samples was higher than that in white bread, which may also explain the elevated vitamin D_2_ levels found in the UV-C-treated whole wheat bread samples.

Moreover, the synthesis of vitamin D_2_ may also be affected by the pore structure of the bread samples. Whole wheat bread has a greater number of larger pores compared to white bread (Figure 2). This abundant porous structure could have facilitated better penetration of UV-C radiation into the interior surface of the whole wheat bread, resulting in increased UV-C exposure and, consequently, a higher conversion of ergosterol to vitamin D_2_.

**Figure 2:**
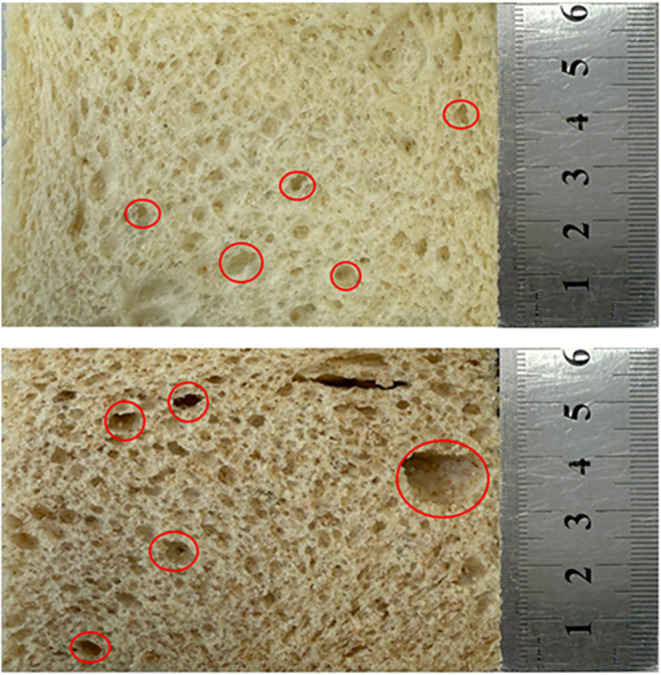
Pore structures of the white (top) and whole wheat bread (bottom) samples.

The recommended dietary allowance (RDA) of nutrients varies based on age and gender. To prevent vitamin D deficiency, a daily intake recommendations are as follows: infants in their first year should receive 10 μg (400 IU), individuals aged 1–70 should get 15 μg (600 IU), and those over 70 years old should aim for 20 μg (800 IU) of vitamin D.^27^ To help individuals meet these daily vitamin D requirements, several countries have implemented mandatory food fortification strategies. In Finland, liquid dairy products are fortified with 1 μg of vitamin D per 100 kcal, and fat spreads contain 20 μg per 100 g.^28^ Similarly, in Sweden, milk is fortified with 0.95–1.10 μg per 100 g, while margarine contains 19.5–21 μg per 100 g, and fermented dairy products and plant-based alternatives are typically fortified with 0.75–1.10 μg per 100 g.^29^ In the U.K., research showed that fortifying bread with 10 μg of vitamin D per 100 g of wheat flour was more effective in improving vitamin D status than fortifying milk, which ranged from 0.25 to 7 mg per 100 L. A modeling study suggested that fortifying bread with 6.25–8.75 μg (250–350 IU) of vitamin D per 100 g would be sufficient to enhance the population’s vitamin D levels. In addition, a randomized controlled trial investigated the effect of vitamin D_2_-fortified bread on serum 25­(OH)­D levels. Daily consumption of bread containing 25 μg of vitamin D_2_ per 50 g led to a significant increase in total serum 25­(OH)­D, with results comparable to supplementation.^30^

Table 3 summarizes the vitamin D_2_ levels quantified in the bread samples, expressed on a fresh weight basis, along with the percentage of RDA provided by a single slice. The percentage of RDA values in Table 3 is based on the recommended intake of 15 μg/day for individuals aged 1–70 years. Consuming UV-C-treated bread can contribute to daily vitamin D intake. A slice of the UV-C treated white bread measuring 6 cm × 9 cm × 1.25 cm (width × length × thickness) can provide 14–33% of the RDA value, whereas a slice of the UV-C-treated whole wheat bread can provide 19–50%, depending on the UV-C dose and dose rate. In our study, the weight of the white bread slice with dimensions of 6 cm × 9 cm × 1.25 cm (width × length × thickness) was about 14 g, and that of the whole wheat bread was 15.5 g. Thus, the amount of UV-C-treated bread consumption required to fully meet the RDA of vitamin D ranges from 42.9 to 100.0 g for white bread, and 31.3 to 83.3 g for whole wheat bread.

**Table 3:** Effect of UV-C Treatments on the Vitamin D_2_ Content (μg/g Based on Fresh Weight) of the Bread and the Contribution of a Slice of Bread^a^ to RDA (%)^b^

dose (kJ/m^2^)	dose rate (kJ/m^2^·min)	white bread	whole wheat bread
0 (control)	0.00	<0.11^e^ (0%)	<0.11^e^ (0%)
0.50	0.03	0.25^c^ (23.3%)	0.29^c^ (30.0%)
	0.13	0.15^d^ (14.0%)	0.18^d^ (18.6%)
2.00	0.03	0.35^a^ (32.6%)	0.48^a^ (49.6%)
	0.13	0.29^b^ (27.1%)	0.36^b^ (37.2%)

a Bread slices (6 cm × 9 cm × 1.25 cm; *w* × *l* × *t*) weighed 14 g (white) and 15.5 g (whole wheat).

b Values with different lowercase letters in the same column are significantly different (*p* < 0.05).

These contributions of UV-C-treated bread to RDA of vitamin D are significantly higher than those found in most commercially fortified products. Modeling studies have suggested bread fortification levels should be between 6 and10 μg per 100 g, while intervention trials have tested doses as high as 50 μg per 100 g, which support the efficacy and safety of higher levels of vitamin D. Accordingly, the vitamin D_2_ content of our treated bread falls within ranges that are both safe and effective, supporting their use as a practical and scalable strategy for increasing vitamin D intake through a commonly consumed staple food.

### 3.2 Moisture Content

The moisture levels of the white and whole wheat bread samples are listed in Table 4. The UV-C treatment did not affect the moisture content of the bread samples (*p* > 0.05). However, it was observed that whole wheat bread had a higher moisture content compared to white bread (*p* < 0.05).

**Table 4:** Effect of UV-C Treatment on the Moisture Content (%) of the Bread Samples^a^

dose (kJ/m^2^)	dose rate (kJ/m^2^·min)	white bread	whole wheat bread
0 (control)	0.00	39.21 ± 1.35^a^	42.44 ± 1.42^a^
0.50	0.03	39.80 ± 1.54^a^	43.20 ± 1.38^a^
	0.13	40.65 ± 1.58^a^	43.43 ± 1.28^a^
2.00	0.03	38.86 ± 1.33^a^	41.73 ± 2.20^a^
	0.13	40.61 ± 1.82^a^	43.26 ± 1.37^a^

a Values with the same letters within the same column are not statistically different (*p* > 0.05). Values are the means ± standard deviations of three observations..

While the optimal moisture content for vitamin D_2_ yield in mushrooms has been investigated, the effects of UV treatment on moisture content have not been extensively studied. However, a study investigated the impact of UV-C treatment on remilled semolina, analyzing changes in its moisture content.^31^ In this study, the semolina was irradiated with UV-C (254 nm) light source of 30W at a distance of 15 cm for durations ranging from 5 to 120 min. A significant decrease in moisture content was observed after the 30 min of exposure, attributed to increased temperature resulting from prolonged UV irradiation, which caused moisture loss through evaporation. In contrast, we found in our study that the applied UV-C dose and dose rate did not significantly affect the moisture content. This may be because the lower UV-C energy used in our study was insufficient to cause moisture loss.

### 3.3 Textural Properties

Changes in the firmness values of white and whole wheat breads are given in Table 5.

**Table 5:** Effect of UV-C Treatment on Firmness (N) of the Bread Samples^a^

dose (kJ/m^2^)	dose rate (kJ/m^2^·min)	white bread	whole wheat bread
0 (control)	0.00	1.82 ± 0.14^a^	2.78 ± 0.16^a^
0.50	0.03	1.65 ± 0.13^ab^	2.52 ± 0.25^ab^
	0.13	1.55 ± 0.11^b^	2.35 ± 0.19^b^
2.00	0.03	1.78 ± 0.13^ab^	2.71 ± 0.17^ab^
	0.13	1.69 ± 0.11^ab^	2.60 ± 0.27^ab^

a Mean values with different lowercase letters in the same column are significantly different (*p* < 0.05). Values are the means ± standard deviations of five observations.

The UV-C dose had a significant impact on the firmness of both white (*p* = 0.026) and whole wheat bread (*p* = 0.043) samples. Dose rate, on the other hand, did not cause a significant change in the firmness of the bread samples (*p* > 0.05). The overall interaction of the dose and dose rate was not significant (*p* > 0.05). Bread samples treated with a 0.50 kJ/m^2^ dose at the high-dose rate exhibited lower firmness than the control samples, while the other treated samples showed firmness levels similar to those of the controls.

Prolonged exposure of bread samples in the chamber, whether UV-C treated or not, may have contributed to surface moisture loss and a potential increase in the crumb firmness values. Notably, the control samples were kept in the chamber for the longest duration (64 min, equivalent to a high dose at the lower dose rate exposure) without UV-C lamps on. These results suggest that extended time spent in the chamber facilitates internal moisture redistribution within the crumb, accelerating starch retrogradation and leading to higher firmness values.^32^ Conversely, the lower firmness observed after just 3.5 min of exposure to the 0.5 kJ/m^2^ dose at the higher dose rate could be attributed to the limited exposure time, which minimized moisture migration and retrogradation. Therefore, the reduction in firmness for samples treated at the low dose may not be exclusively due to UV-C treatment but rather a combination of reduced moisture loss and slower staling kinetics.

Furthermore, whole wheat bread had higher firmness than white bread (*p* < 0.05). In the production of whole wheat bread, sourdough powder was used, differing from the production of white bread. The inclusion of sourdough powder likely contributes to a more elastic gluten structure and a porous crumb texture with thicker cell walls, resulting in a firmer bread crumb.^33^

### 3.4 Color Properties

The effects of UV-C treatment on the color properties of bread crumbs were evaluated using L*, a*, b*, and Δ*E* values, as presented in Table 6. The Δ*E* values were calculated in comparison to those of the control samples. There was no significant difference in the Δ*E* values for either white or whole wheat bread after UV-C treatment (*p* > 0.05). Similarly, the L*, a*, and b* values remained statistically unchanged following UV-C exposure for both bread types.

**Table 6:** Effect of UV-C Treatment on Color Properties of the Bread Samples^a^

	dose (kJ/m^2^)	dose rate (kJ/m^2^·min)	L*	a*	b*	Δ*E*
white bread	0.50	0.03	80.43 ± 0.52^a^	0.79 ± 0.29^a^	17.74 ± 0.30^a^	1.66 ± 0.57^a^
		0.13	79.80 ± 0.34^a^	0.85 ± 0.04^a^	17.93 ± 0.29^a^	1.08 ± 0.39^a^
	2.00	0.03	79.81 ± 1.19^a^	0.86 ± 0.37^a^	18.40 ± 0.51^a^	1.15 ± 0.96^a^
		0.13	80.33 ± 0.24^a^	0.89 ± 0.34^a^	18.05 ± 0.72^a^	1.46 ± 0.57^a^
whole wheat bread	0.50	0.03	64.81 ± 0.91^A^	5.32 ± 0.43^A^	19.79 ± 0.39^A^	1.44 ± 0.70^A^
		0.13	63.84 ± 0.95^A^	5.55 ± 0.37^A^	20.28 ± 0.24^A^	1.00 ± 0.20^A^
	2.00	0.03	64.33 ± 0.90^A^	5.36 ± 0.41^A^	20.34 ± 0.90^A^	1.28 ± 0.49^A^
		0.13	64.82 ± 1.07^A^	5.35 ± 0.28^A^	19.88 ± 0.86^A^	1.66 ± 0.62^A^

a Values with the same letters within the same column are not statistically different (*p* > 0.05); lowercase letters indicate comparisons within white bread, and uppercase letters indicate comparisons within whole wheat bread. Values are the means ± standard deviations of five observations.

UV treatment has been reported to affect surface color in food by initiating chemical reactions that alter the pigment stability or promote browning. For instance, prolonged UV-C (254 nm) exposure on cake batter before baking resulted in surface browning, evidenced by decreased L* and b* values and increased a* values.^34^ Conversely, we did not find any significant color changes following UV-C treatments, which may be due to the applied dose and dose rate being insufficient to induce visible pigment alterations in the bread crumbs. Color differences with Δ*E* values below 2 are generally not visually perceptible by human eyes and may only be noticeable through close inspection.^35^ In our study, Δ*E* values for white and whole wheat bread remained below 2, indicating that the observed color changes are minimal and not easily discernible.

In addition to these parameters, chroma and hue values were also investigated, and no significant differences were found after UV-C treatment (*p* > 0.05). The chroma values ranged from 17.7 to 18.7 for white bread and 20.4 to 21.0 for whole wheat bread. The hue values range from 87.4 to 87.7 for white bread and 74.9 to 75.7 for whole wheat bread. Chroma defines the color intensity, while hue represents the angle, where 0° corresponds to red, 90° to yellow, 180° to green, and 270° to blue.^36^ The stability of chroma and hue values indicate that UV-C treatment did not significantly affect the color tone or intensity of either bread sample. The hue angle of white bread aligns more closely with a yellow tone, whereas whole wheat bread tends toward a red tone. This difference can be attributed to the presence of wheat bran, which contributes to a darker, more saturated crumb color and reduces the hue angle in whole wheat bread.^37^

The statistically insignificant differences in Δ*E*, chroma, and hue values indicate that the UV-C treatment did not affect the bread color. This finding aligns with the sensory evaluation results, which confirmed comparable color homogeneity in the crumb of both UV-C-treated and control bread.

### 3.5 Sensory Properties

The UV-C treatments had some impact on the sensory attributes of the bread samples, as summarized in Figure 3.

**Figure 3:**
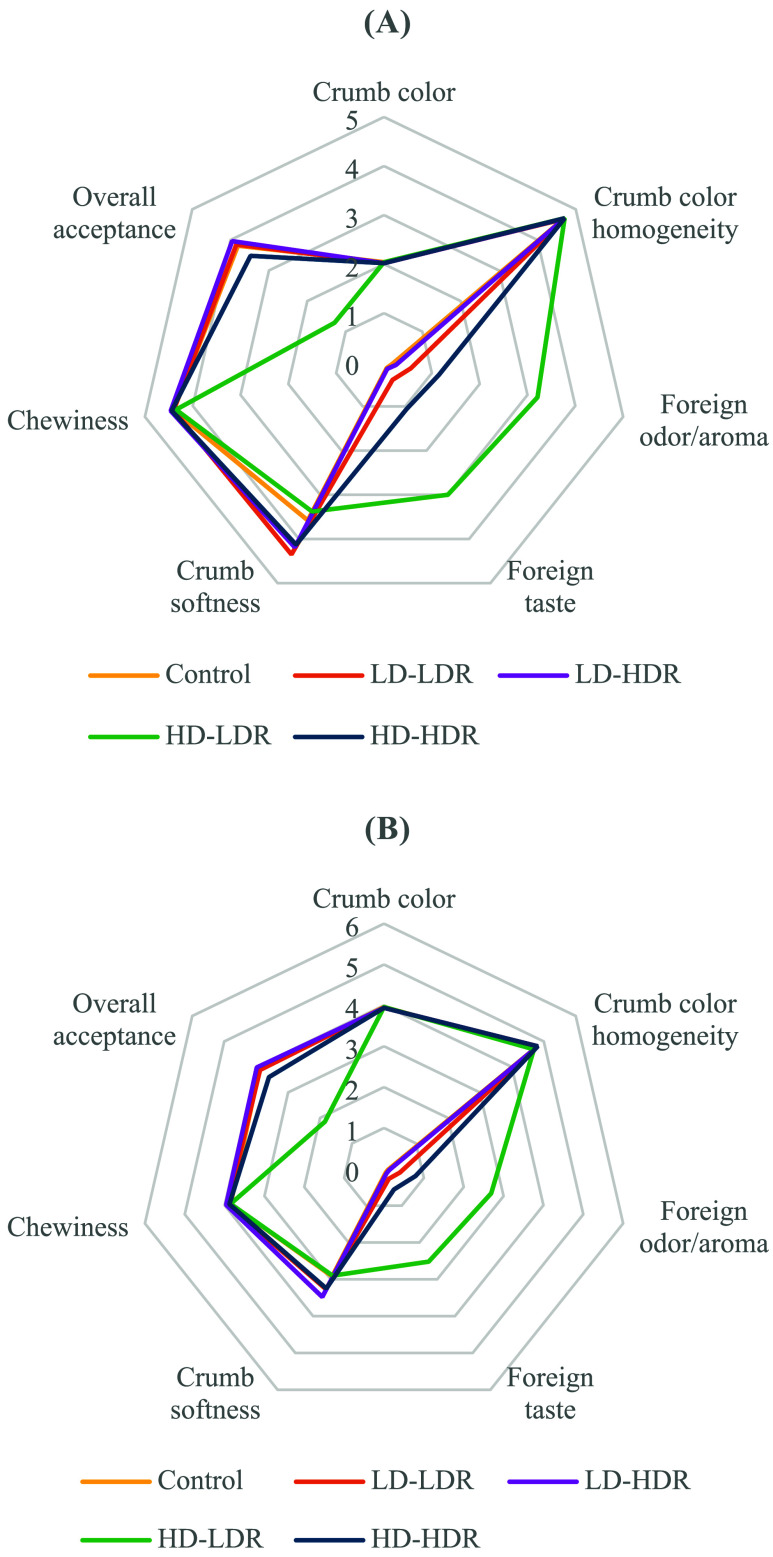
Effect of UV-C treatment on the sensory properties of white (A) and whole wheat (B) bread samples. LD-LDR: low dose (0.5 kJ/m^2^) at low-dose rate (0.03 kJ/m^2^·min), LD-HDR: low dose (0.5 kJ/m^2^) at high-dose rate (0.13 kJ/m^2^·min), HD-LDR: high dose (2.0 kJ/m^2^) at low-dose rate (0.03 kJ/m^2^·min), HD-HDR: high dose (2.0 kJ/m^2^) at high-dose rate (0.13 kJ/m^2^·min).

In terms of crumb color, no significant difference was found between the UV-C-treated bread samples and the control samples (*p* > 0.05). The uniformity of the crumb color remained high and was not affected by the treatments (*p* > 0.05). Therefore, the UV-C treatments did not result in any detectable changes in sensory color of the bread samples, which aligns with the results obtained from color analysis.

However, the UV-C dose and dose rate significantly affected the foreign odor/aroma and foreign taste of both types of bread samples (*p* < 0.05). As the UV-C dose increased and the dose rate decreased, the scores for these attributes also rose. The interaction between dose and dose rate also had a significant effect on the foreign odor/aroma and taste of the bread samples (*p* < 0.05). The effect of dose rate was particularly noticeable at a dose of 2.00 kJ/m^2^ compared to 0.50 kJ/m^2^, where a lower dose rate at 2.00 kJ/m^2^ led to a higher increase in scores. The highest scores for foreign odor/aroma, 3.21 and 2.69, were observed in white and whole wheat bread samples, respectively, when treated with a dose of 2.00 kJ/m^2^ at a low-dose rate of 0.03 kJ/m^2^·min (HD-LDR). Under the same treatment conditions, the foreign taste scores were 3.0 for white bread and 2.5 for whole wheat bread. In the sensory analysis, a score of 2, indicating “distinct”, is the maximum acceptable threshold for foreign odor/aroma and foreign taste. Thus, HD-LDR treatment resulted in scores exceeding this threshold, showing a potential rejection of the samples. In contrast, the other treatment conditions did not result in undesirable levels of foreign odor, aroma, or taste, making those samples acceptable.

UV-C treatments can cause oxidative reactions in treated foods, which may affect their sensory properties. Kawaguchi et al.^38^ investigated the effect of UV-C (254 nm) treatment on mold control in white bread production and its impact on sensory quality. Their results showed that UV-C dose at 70 mJ/cm^2^ (equivalent to 0.70 kJ/m^2^) effectively delayed mold growth on white bread without affecting taste, while higher doses (140 and 210 mJ/cm^2^) caused undesirable changes like bitterness and a “plastic-like” flavor. Although they did not investigate different dose rates for the same UV-C dose, their findings align closely with those of our study. In our research, we did not detect any unpleasant taste with a UV-C dose of 0.50 kJ/m^2^ at both dose rates and a dose of 2.00 kJ/m^2^ applied at a high-dose rate. However, a foreign taste was detected in the HD-LDR treatment, which had the longest exposure time. Researchers treated cake batter before baking with UV-C at an intensity of 3.636 mJ/m^2^·s for up to 4 h of exposure and found that the treatments negatively impacted sensory attributes on the cake surface, with decreased taste and odor scores compared to the control.^34^ In our study, although longer durations (64 min) in HD-LDR treatment led to statistically higher foreign taste and odor/aroma scores for bread samples compared to the control, no such effect was observed with the other treatment conditions. The lower dose used in our study may account for the differences that we observed.

In the sensory evaluation of bread crumb softness, both the UV-C dose and dose rate had a significant effect (*p* < 0.05). Treatment with a dose of 0.50 kJ/m^2^ resulted in higher sensory softness scores compared to both the 2.00 kJ/m^2^ dose and the control group. Conversely, a reduction in dose rate led to a decline in sensory softness score. The firmness and the sensory softness of the samples were similarly affected by the treatments.

The softness score, defined by the force needed to compress the bread between two fingers, ranges from 0 (’not soft’) to 5 (“very soft”), with lower scores indicating a decrease in sensory softness. The samples treated with HD-LDR had similar softness scores to the control samples; however, both the control and HD-LDR samples exhibited significantly lower scores (*p* < 0.05) compared to other treatments. The sensory softness of bread samples subjected to prolonged exposure, whether treated with UV–C or not, was lower than the other samples. However, the magnitude of this difference was minimal, with an average score reduction of 17.5% for white bread and 12.8% for whole wheat bread. This could be attributed to the time spent in the chamber during treatment, as the control and HD-LDR samples stayed in the chamber for 64 min, but the other samples had shorter exposure times. Texture analysis revealed that only the LD-HDR treatment (which lasted 3.5 min) resulted in a significantly lower firmness, meaning higher softness. In the sensory analysis, however, samples treated for 3.5 and 15 min (LD-LDR, LD-HDR, and HD-HDR) were rated by the panelists as statistically similar in sensory softness, while both were perceived softer than the samples exposed for 64 min (control and HD-LDR) treatments.

In evaluating chewiness, which refers to the ease of chewing, the effects of the applied UV-C dose and dose rate were not significant (*p* > 0.05). Chewiness scores range from 0 (“not chewable”) to 5 (“very easy to chew”), with lower scores indicating reduced chewiness. The UV-C treatment did not negatively impact the chewiness properties of the bread samples. Since UV-C radiation has a relatively low penetration depth on food surfaces, it primarily affects surface characteristics rather than the bulk textural properties.

In the overall acceptance evaluation of the bread samples, however, the UV-C dose and dose rate had a significant effect (*p* < 0.05). Increasing the UV-C dose and decreasing the dose rate led to a reduction in the acceptability of the bread samples. The interaction between dose and dose rate also significantly affected the overall acceptance of the bread samples (*p* < 0.05). While no significant change in overall acceptability was observed with reducing dose rates at the 0.50 kJ/m^2^ dose, a notable decrease in overall acceptance was observed when the dose rate was reduced at the 2.00 kJ/m^2^ dose. The overall acceptance scores ranged from 0 (“dislike”) to 5 (“very like”), with lower scores indicating decreased acceptance. A score of 3, indicating ’moderately like’, was considered the minimum acceptable threshold. The HD-LDR treatment of both white and whole wheat breads resulted in scores below 3, which are considered below the threshold of acceptability. The overall acceptance of the bread samples decreased, primarily due to the presence of off-flavor/aroma and off-taste. Other treatments, however, did not result in unacceptable scores for overall acceptability in either white bread or whole bread.

HD-HDR treatment resulted in the highest increase in vitamin D_2_ without adversely affecting sensory attributes, making it the optimal condition. Sensory panel scores showed no significant decline in quality, indicating a favorable balance between the nutritional enhancement and consumer acceptability.

## 4 Conclusions

In conclusion, the findings of this study clearly demonstrate that the UV-C treatment significantly increases vitamin D_2_ levels in both white bread and whole wheat bread. While a UV-C dose of 2.00 kJ/m^2^ at a dose rate of 0.03 kJ/m^2^·min (HD-LDR) negatively affected sensory properties, other dose and dose rate combinations (HD-HDR, LD-LDR and LD-HDR) allowed for significant vitamin D_2_ enrichment without compromising the quality of bread. The optimal condition identified was a UV-C dose of 2.00 kJ/m^2^ at a dose rate of 0.13 kJ/m^2^·min, which provided the highest vitamin D_2_ without adversely affecting the bread’s quality. These findings highlight the potential of UV-C as a practical postbaking vitamin D fortification method to deliver nutritionally relevant amounts of vitamin D_2_ through bread, a widely consumed food product. Regular consumption of UV-C-treated bread can meaningfully contribute to daily dietary vitamin D intake.

Further investigations are necessary to explore how the concentration of ergosterol, a precursor to vitamin D_2_, in bread may affect its UV-C-induced formation. Besides, the impact of the UV-C treatment on microbial stability and the stability of vitamin D_2_ in bread during storage requires further study. Research is also needed to examine the bioavailability of vitamin D_2_ from UV-treated bread.
